# The synergistic role of sulfuric acid, ammonia and organics in particle formation over an agricultural land†

**DOI:** 10.1039/d3ea00065f

**Published:** 2023-07-07

**Authors:** Lubna Dada, Magdalena Okuljar, Jiali Shen, Miska Olin, Yusheng Wu, Laura Heimsch, Ilkka Herlin, Saara Kankaanrinta, Markus Lampimäki, Joni Kalliokoski, Rima Baalbaki, Annalea Lohila, Tuukka Petäjä, Miikka Dal Maso, Jonathan Duplissy, Veli-Matti Kerminen, Markku Kulmala

**Affiliations:** a Institute for Atmospheric and Earth System Research, University of Helsinki PO Box 64, 00014 Finland lubna.dada@helsinki.fi markku.kulmala@helsinki.fi; b Laboratory of Atmospheric Chemistry, Paul Scherrer Institute 5232 Villigen Switzerland; c Aerosol Physics Laboratory, Tampere University PO Box 692, 33014 Tampere University Finland; d Finnish Meteorological Institute PO Box 503 00101 Helsinki Finland; e Qvidja Research Farm Qvidja 15 21630 Parainen Finland; f Helsinki Institute of Physics (HIP)/Physics, Faculty of Science, University of Helsinki 00014 Helsinki Finland

## Abstract

Agriculture provides people with food, but poses environmental challenges. *Via* comprehensive observations on an agricultural land at Qvidja in Southern Finland, we were able to show that soil-emitted compounds (mainly ammonia and amines), together with available sulfuric acid, form new aerosol particles which then grow to climate-relevant sizes by the condensation of extremely low volatile organic compounds originating from a side production of photosynthesis (compounds emitted by ground and surrounding vegetation). We found that intensive local clustering events, with particle formation rates at 3 nm about 5–10 times higher than typical rates in boreal forest environments, occur on around 30% of all days. The requirements for these clustering events to occur were found to be clear sky, a low wind speed to accumulate the emissions from local agricultural land, particularly ammonia, the presence of low volatile organic compounds, and sufficient gaseous sulfuric acid. The local clustering will then contribute to regional new particle formation. Since the agricultural land is much more effective per surface area than the boreal forest in producing aerosol particles, these findings provide insight into the participation of agricultural lands in climatic cooling, counteracting the climatic warming effects of farming.

## Abstract 2

Agricultural lands produce high loads of aerosol particles, which could cool the climate, overcoming the well-known climate warming effects of agriculture.

## Graphical abstract

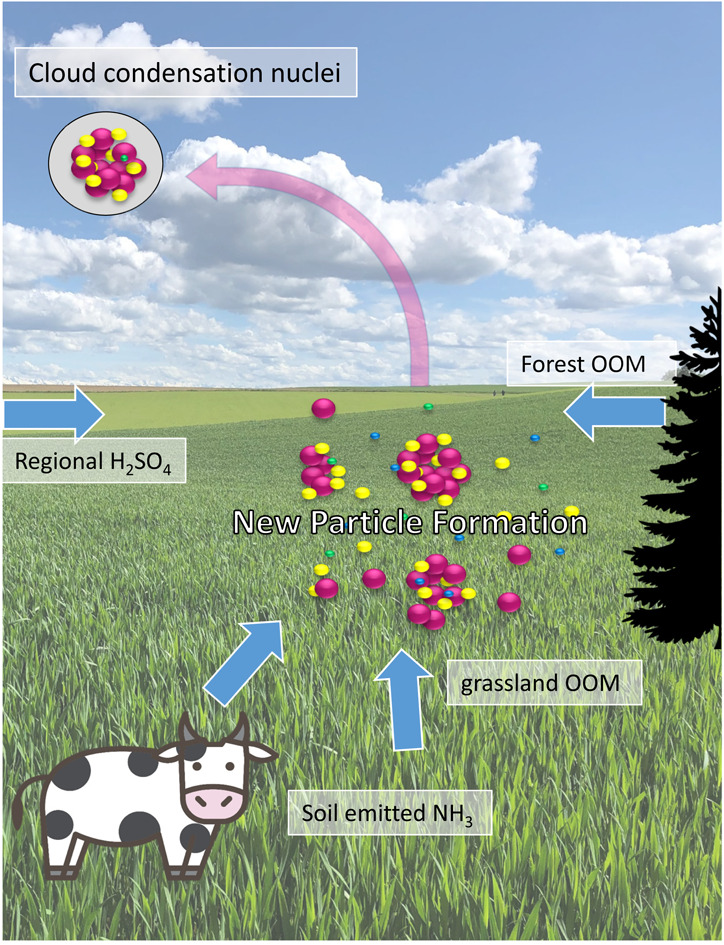

**Environmental significance statement**

Agriculture is recognized for its undesirable effects on our climate due to greenhouse gas and ammonia emissions. However, agriculture remains the main source of food to humankind. In this study, *via* comprehensive observations on an agricultural land at Qvidja in Southern Finland, we were able to show that soil-emitted compounds, together with available sulfuric acid, form new aerosol particles, which then grow to climate-relevant sizes by the condensation of extremely low volatile organic compounds originating from a side production of photosynthesis. We find that agricultural land areas are more than 10 times more efficient in producing growing aerosol particles than the adjacent boreal forest environment, and hence are expected to be several times more efficient in eventually producing cloud condensation nuclei. Given the role of aerosols in cooling the climate, our findings are of unique importance for implementation in global models not yet accounting for the contribution of agricultural lands to the global aerosol budget. Finally, *via* introducing regenerative farming processes to agricultural lands enabling them to become efficient carbon sinks, together with the capability of such lands of producing large aerosol particle concentrations, the recognition of agricultural lands as solely global warming contributors could change.

## Introduction

The global environmental grand challenges, including climate change, food production, water supply and biodiversity, are tightly linked to each other.^1^ Also, biogeochemical cycles of carbon, nitrogen and water as well as the physics and chemistry of atmospheric aerosols and atmospheric chemistry are interlinked.^2^ In order to overcome these grand challenges, we need to understand these connections and interlinks. Modern industrialized agriculture is thought to be on one hand crucial for human food supply but at the same time, it poses challenges for the climate due to greenhouse gas and ammonia emissions, as well as for biodiversity due to monoculture.^3^ Therefore, a shift in paradigm is needed in order to neutralize the harmful climatic effects of agriculture.

Atmospheric aerosol particles, in addition to deteriorating human health,^4^ have profound impacts on the Earth-atmosphere continuum, including weather, climate, air quality and ecosystems.^5^ Aerosol–radiation and aerosol–cloud interactions give large contributions to uncertainties in climate change predictions.^5^ The dominating number fraction of aerosol particles is formed *via* gas-to-particle conversion during atmospheric new particle formation (NPF) events.^6^ These events take place almost everywhere, from relatively clean environments like boreal forests to rather polluted environments like low and middle income megacities.^7–9^ NPF involves clustering of precursor vapors, usually sulfuric acid stabilized with bases (ammonia or amines), and subsequent growth by condensing vapors, which are typically low volatile organic and inorganic compounds.^10^ The critical cluster size regime of nucleation is commonly considered to be in the range of 1.5–2 nm.^11^ From an observational point of view, there is usually a burst of new atmospheric clusters in the sub-3 nm size range, followed by their subsequent growth to larger sizes.

The interactions and feedback between surface-emitted gases and formed aerosol particles have been discussed by Kulmala *et al.*,^12^ who proposed a continental biosphere–aerosol–cloud–climate (COBACC) feedback mechanism connecting aerosol particles, photosynthesis, aerosol–radiation and aerosol–cloud interactions, and climate. Gross Primary Production (GPP) which is the gross amount of carbon dioxide (CO_2_) fixed by primary producers through photosynthesis, is one of the most important characteristics describing the ecosystem's functionality. The overall activity of the primary producers, mainly plants in terrestrial ecosystems, is influenced by several environmental factors, such as the temperature, local hydrology and light in the form of photosynthetically active radiation (PAR). Depending on soil properties, soil and crop management practices, such as tilling, sowing, harvesting and fertilization, can produce precursor gases that may have direct effects on NPF.^13^ For instance, organic waste products, which are usually applied to cropland as fertilizers, are found to be a major source of NPF, based on chamber measurements.^14^ Similarly, land management may promote or hinder primary production^15^ and cause changes in the ecosystem functions that indirectly influence NPF or growth of the newly formed particles. While agriculture contributes to primary aerosol emissions, there are currently no estimates of NPF or secondary organic aerosol formation from gaseous precursors from agriculture.^14^ On a global scale, agricultural lands cover approximately the same area as forests,^16,17^ making their potential influences on atmospheric processes substantial. However, measurements inside crop fields or agricultural lands remain rare,^18–20^ with limited information on the vapors responsible for particle formation and growth, making the importance of a large fraction of earth's surface in aerosol formation unknown.

In this work, to assess the contribution of agricultural lands to atmospheric processes including NPF events, we deployed a comprehensive suite of instrumentation, including gas and flux monitors, cluster composition measurement spectrometers and particle size distribution spectrometers in Qvidja, Southern Finland. The studied field is a grassland, and the soil type is clay, representing a typical northern hemisphere agricultural land.^21^*Via* these measurements, we investigate the precursors driving the formation of the smallest particles, as well as the conditions which inhibit their formation. In addition, we study the composition and the role of regionally emitted organic vapors in growing the freshly formed particles where they can contribute to regional new particle formation. All in all, we show that agricultural lands are capable of producing high particle concentrations (and thus high cloud condensation nuclei concentrations) compared with the boreal forest, and when combined with their carbon sink potential, they are expected to have a cooling effect on the climate that compensates for a big fraction of the warming effect associated with agriculture.

## Materials and methods

### Measurement sites

#### Qvidja site

The Qvidja farm in Parainen (southwest Finland), referred to as Qvidja hereafter, is located near the coast of Finland and comprises a permanent grassland, a small scale crop production, as part of experimental work on organic amendments, and pastures used for horse care. The measuring station was established at the edge of the investigated grassland area (60°17′43.8′′ N, 22°23′34.1′′ E). The exact location may be seen from Fig. S1.† The largest city near the farm is Turku with 191 000 inhabitants, located about 25 km northwest inland.

Qvidja is a pilot farm for regenerative farming and flagship for Carbon Action platform, initiated by the owners I. Herlin and S. Kankaanrinta. The farming soils of Qvidja are mostly clay loams. These fields are now being upgraded with multi-species, which keeps living roots alive all year because the soil is covered. In the years 2019 and 2020, the grassland has had 9–14 pasture species. In an effort to restore natural processes to their natural condition, existing grassland is supplemented with a wide variety of grasses, forbs, legumes, herbs, and even woody plants. Grazing pressure and rest periods are also managed. The growth season begins in late April and lasts until the end of October. Precipitation ranges between 500 and 700 mm every year. The growing season receives about 350 mm of precipitation, but the majority of it arrives late in the season, making the main production extremely dependent on soil water storage. See Nevalainen *et al.*^22^ and Heimsch *et al.*^15^ for more details on soil carbon sequestration and effects of carbon farming practices in Qvidja.

Horses, cattle and sheep graze on Qvidja's lands. Animals are necessary for biodiversity and farm-specific functionality, as part of the field-pasture ecosystem in Qvidja. Qvidja's fields are only tilled if necessary, to help establish a healthier plant community and biological function. Tillage is applied in an appropriate way only to the very top layers of soil to save worms, mycorrhizal fungi and other soil biology. Qvidja does not use pesticides, herbicides, fungicides or insecticides. Fertilizing is mainly organic, and in May it contained a mixture of side products from industries of starch potato processing, biowaste processing and ethanol production out of sawdust. This fertilization mixture contained 70% (of dry weight) of organic matter, 1.3% of nitrogen, 0.2% of phosphorus, 3% of potassium and 0.4% of sulfur, as well as small amounts of calcium, magnesium, zinc, copper and manganese. In 2019, approximately 4600 kg ha^−1^ of this mixture was applied to the field on 8 May. On 26 June after the first harvest, 220 kg ha^−1^ of mineral fertilizers were added. This fertilizer contained 23% of nitrogen, 10% of phosphorus and 8% of potassium. The management activities, during 2019, are summarized in Table S1.† The grass was harvested twice during the growing season of 2019: first in June (11.6.) and again in August (20.8.).

Regenerative agriculture practices have been recently implemented at Qvidja and their long-term effects will be assessed in future studies. These farming and grazing practices can, among other benefits, reverse climate change by rebuilding soil organic matter and restoring degraded soil biodiversity which results in both carbon drawdown and improves the water cycle. Previously, dedicated studies at the site have shown that the field acts as a net carbon sink with a net carbon balance of −86 ± 12 g cm^−2^ per year between 4th May 2019 and 3rd May 2020.^15^

#### SMEAR II station at Hyytiälä

The SMEAR II station (Station for Measuring Ecosystem–Atmosphere Relations) is located in Hyytiälä (61.1° N, 24.17° E; 181 m a.s.l.), Southern Finland and compromises comprehensive observations of trace gases, particle measurements and auxiliary measurements as early as 1995.^23^

### Instrumentation

#### Particle and ion number size distributions

Aerosol particle size distributions between 1 nm and 1 μm were obtained by combining measurement from three instruments: Particle Size Magnifier (PSM^24^), Neutral cluster and Air Ion Spectrometer (NAIS^25^) and Differential Mobility Particle Sizer (DMPS^26^). The PSM activates and grows smallest particles up to 90 nm using diethylene glycol as a working fluid, after which they are counted using a Condensation Particle Counter (CPC).^27^ The saturation flow rate of the PSM is varied to measure a particle size distribution 1 nm and 3 nm. The kernel method was used to invert the PSM data from counts to particle number size distribution and then line loss corrections were applied.^28^

The NAIS measures the particle number size distribution between 0.8 and 40 nm for atmospheric naturally charged particles and between 2.5 and 42 nm for total (naturally charged + neutral particles). A home-built DMPS was used to measure the particle number size distribution between 6 nm and 1 μm. The DMPS is maintained and regularly calibrated for sizing accuracy and total particle concentration following the standard operation procedure by Wiedensohler *et al.*^29^ On the other hand, the NAIS requires a comparison to a reference instrument when considering absolute particle concentrations. The NAIS is expected to overestimate the particle concentration by up to a factor of 10 compared to the DMPS.^30^ Here, *via* comparing to the DMPS (ratio of DMPS to NAIS) in the overlapping size range 6–42 nm, a correction factor for the NAIS can be derived. The NAIS was found to overestimate the concentration by a factor 5, which is the factor used to correct the NAIS concertation prior to the combination of the size distributions from the three instruments (Fig. S2†). The same instrumentation (NAIS and DMPS) was also available in Hyytiälä and the data were combined in the same way as in Qvidja for comparison.

#### Trace gases and fluxes

At Qvidja, the net CO_2_ exchange (NEE) was measured using the eddy covariance method.^15^ The fast-response instruments for measuring fluctuations of CO_2_ concentration and vertical wind speed were located in a mast at a height of 2.3 m. Net half-hourly CO_2_ flux is obtained as a covariance between variations of vertical wind and gas concentration from high-frequency data. The obtained flux typically represents a few hectares area upwind from the measurement point. GPP was obtained from the measured NEE by using simple temperature and radiation response functions. First, an exponential temperature response function was fitted to NEE measurements during night (PAR <20 μmol m^−2^ s^−1^), which represent ecosystem respiration, by using both soil and air temperature. From that function, respiration was estimated for each half-hour. After that, a radiation response function was fitted to measured NEE and calculated respiration, and by using the fitted parameters from that function, GPP could be solved for each half-hour by using the measured PAR values. O_3_ concentrations were obtained from the Utö Atmospheric and Marine Research Station of Finnish Meteorological Institute, located on Utö Island (59° 46′50 N, 21° 22′23 E, 8 m a.s.l.) at the outer edge of the Archipelago Sea, which is located *ca.* 80 km southwest of Qvidja.^31^

#### Mass spectrometry measurements

In this study, two nitrate-ion-based chemical ionization mass spectrometers were used to measure gas molecules in Qvidja. One spectrometer was coupled with a chemical ionization Eisele-type inlet that is similar to one described by Eisele and Tanner^32^ (NO_3_^−^-CIMS). This instrument was calibrated with a general calibration coefficient which is determined from calibration with sulfuric acid, *C*_H_2_SO_4__ = 5.02×10^9^ cm^−3^ per normalized signal (cps cps^−1^; cps, signifies counts per second). The detailed description of the instrument and calibration method can be found in Jokinen *et al.*^33^ and Kurten *et al.*,^34^ respectively. The instrument was run continuously from the beginning of April to the end of June 2019. After that period, we deployed another mass spectrometer coupled with a Multi-scheme chemical IONization inlet (NO_3_^−^-MION-CIMS)^35^ to cover the rest of the measurement campaign. Rissanen *et al.*,^35^ Wang *et al.*,^36^ and Huang *et al.*^37^ described this inlet in detail for its chemical ionization method, inlet design, setup, and operation. The design of the MION inlet is highly beneficial for ion combinations, *e.g.*, NO_3_^−^ and Br^−^, which enables significantly increased chemical information obtained from different chemical ionization. In this study, we measure oxygenated organic molecules (OOMs),^38^ sulfuric acid (H_2_SO_4_),^33^ iodic acid (HIO_3_),^39^ and methane sulfonic acid (MSA), which work well with adduct-forming reagent ions (*e.g.*, NO_3_^−^). This instrument was operated by the University of Helsinki from May to December 2019, but it had not been working all the time due to power failure and short circuits in the inlet caused by anion accumulation from ammonium nitrate. The quantitative measurements of this instrument rely on the inter comparison of H_2_SO_4_ concentration with the pre-calibrated NO_3_^−^-CIMS. As shown in Fig. S3,† the linear fit between NO_3_^−^-CIMS and NO_3_^−^-MION-CIMS for the overlap time (13–23 June 2019) shows that the H_2_SO_4_ calibration coefficient in NO_3_^−^-MION-CIMS is around three times larger than that of NO_3_^−^-CIMS. This is likely due to the different sensitivity between the two instruments. Hence, we applied another sulfuric acid calibration coefficient, *C*_H_2_SO_4__ = 1.6×10^10^ cm^−3^ per normalized signal, to NO_3_^−^-MION-CIMS. *C*_H_2_SO_4__ is applied to the normalized signals of HIO_3_, MSA, and OOMs as a general calibration coefficient. Analogous to H_2_SO_4_, we assume that HIO_3_, MSA, and OOMs have a collision-limited charging efficiency when reacting with the nitrate ions according to previous studies.^40^ Besides, we apply this calibration coefficient to all the conditions since it has been shown that temperature or humidity does not change the charging efficiency of these technologies significantly.^41^ In Hyytiälä, the sulfuric acid concentration was obtained using a nitrate-ion-based chemical ionization mass spectrometer (NO_3_^−^-CIMS), at a 35 m mast, which was calibrated following the protocol described by Kürten *et al.*^34^ to ensure comparability.

#### Measurement of NH_3_

Gaseous ammonia (NH_3_) concentrations were monitored using an AiRRmonia NH_3_ analyzer. The AiRRmonia was originally developed by ECN (Energy Research Centre of the Netherlands, Petten, NL),^42^ and has been further improved and commercialized by Mechatronics Instruments (b.v., Hoorn, NL).^43^ The instrument uses a Teflon membrane to strip the NH_3_ from the airflow into a deionized water flow, followed by selective ion membrane and conductivity measurement. The instrument's detection limit is 0.1 μg m^−3^ (∼140 pptv).

#### Meteorology

Photosynthetically active radiation (PQS PAR sensor, Kipp & Zonen B.V., Delft, the Netherlands), global and reflected solar radiation (CMP3 radiometer, Kipp & Zonen), and air temperature and relative humidity (Humicap HMP155, Vaisala Oyj) were measured at a height of 1.8 m. The wind speed (WS) was measured using a three-dimensional sonic anemometer (uSonic-3 Scientific, METEK GmbH, Elmshorn, Germany) at a height of 2.3 m.

### Data analysis

#### Classification of local clustering and regional events

**Fig. 1:**
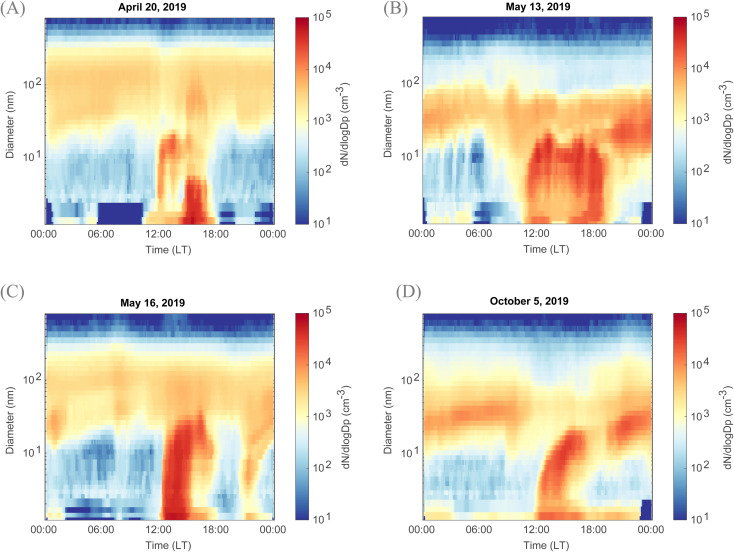
Unique and intense local clustering NPF events in Qvidja. Examples of regional NPF events (A) before, (B) and (C) after fertilization on May 08th and (D) after harvest on August 20th. Events in Qvidja are characterized by their intense particle formation, fast growth and long time span. The events are also observed to start from the smallest size ranges indicative of local clustering.

Days during which a new mode of particles showing signs of growth appears in the particle number size distribution are classified as event days.^44^ This classification applies for the boreal forest environment in Hyytiälä. In Qvidja, the distinction between regional and local clustering NPF events is needed and depends on both (1) the size during which the first particles are formed and (2) their observed growth to larger sizes in the particle number size distribution data. Local clustering events are observed in the smallest size bins (sub-3 nm),^45^ and hence from near-measurement produced precursors (depending on the condensation sink) and exhibit a ‘bump’ or ‘apple-type’ particle number size distribution.^44^ Regional events are known to extend over hundreds of kilometers.^46^ They are not necessarily observed in the smallest size bins (sub-3 nm) as they could be transported to the measurement location.^45,47^ They exhibit a ‘banana-shaped’ particle number size distribution and are sometimes observed to grow up to hundreds in nm.^48^ On some days, local clustering events have limited growth where their end diameter did not exceed a few tens of nm. On other days, local clustering events merge with regional events and together are observed to reach up to several hundreds in nm. In this study, in Qvidja, days were classified (1) as ‘local clustering events’ (LC) when a new growing mode starting from the sub-3 nm range is observed, (2) as ‘regional event days’ (Reg) when the growth of particles was observed but no new particles were observed below the size of 10 nm, and (3) as ‘non-event days’ (NE) when neither new particles in the sub-10 nm range nor a growing nucleation mode was observed. Examples of each of these events are shown in Fig. 1 and S4.† Unclear cases were classified as undefined days and were excluded from further analysis. In comparison, days in Hyytiälä were classified into three categories only events, non-events and undefined days. A direct comparison between events observed in Qvidja and Hyytiälä is presented in Fig. S5.† The frequency of events in each of the two locations in shown in Fig. S6.†

#### Brightness parameter

The brightness parameter (*P*) is defined by the fraction of the total solar radiation reaching the measurement location after being blocked by existing clouds. The parameter is thus calculated as the ratio between measured global radiation (GlobRad) and theoretical maximum (TheoMax) solar radiation at the top of the atmosphere:^49^1
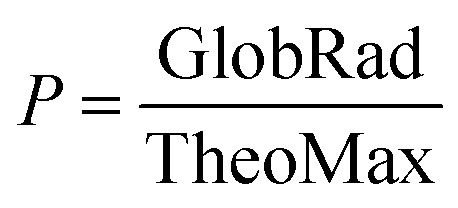


The larger the *P* value, the fewer the clouds in the sky and more radiation penetrates to the ground level. A complete overcast condition is represented by *P* < 0.3 and clear-sky conditions are represented by *P* > 0.7.

#### Particle growth rates

Particle apparent growth rates (GRs) were calculated using the 50% appearance time method using the charged particle number size distribution data measured by the NAIS operated in negative mode.^50,51^ The 50% appearance time method determines the GR of particles as the slope between the diameter of the particles and the time at which 50% of the maximum concentration is reached. In this study, the GRs for the size classes 1.5–3 nm, 3–7 nm and 7–30 nm were calculated. In the cases when the upper diameter 30 nm is not reached, the GR in the last size class is determined for the size class 7-end diameter. In Qvidja, given the nature of local clustering events, it was challenging and in some cases impossible to obtain a GR with an acceptable error, and in other cases, we expect the GR to be underestimated. The growth rates were used in the formation rates' calculation in the next section.

#### Particle formation rates

Particle formation rates at 1.5 nm (*J*_1.5_) were calculated using the balance equation described in Kulmala *et al.*^48^ where the change in the concentration of particles within a certain size bin (here 1.5–3 nm) depends on the particle sources (NPF) and the available sinks (coagulation and growth out of the size bin).2
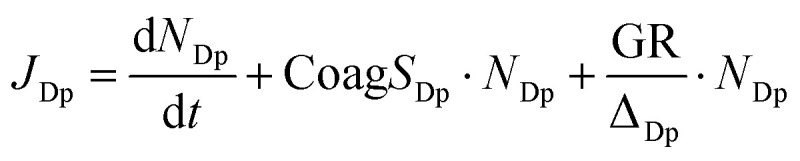


Dp represents the lower diameter of the bin (here 1.5 nm), *N*_Dp_ is the particle number concentration inside the size bin (1.5–3 nm), and GR is the growth rate of particles out of the bin (GR 3–7 nm). Δ_Dp_ is the difference between the upper and lower ends of the size bin of interest (here Δ_Dp_ = 1.5 nm). The GR is calculated as described in the previous section. However, during the events for which a GR could not be obtained given the rapid growth of the particles or the interruption of the growth, we used a median growth rate of all the events in the same month to estimate the formation rate.^52^ To be consistent, a similar approach was used for calculating particle formation rates on non-event days. *J*_3_ and *J*_6_ were calculated in a similar way to *J*_1.5_, using the size bins 3–6 nm and 6–10 nm, respectively.

#### Coagulation and condensation sink

The coagulation sink (Coag*S*) describes the rate at which freshly formed particles of a certain diameter Dp are lost to pre-existing particles as follows:3
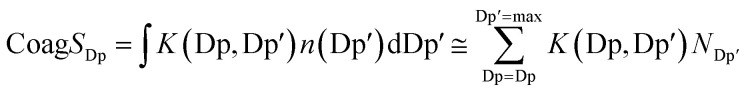


Here, *K* (Dp, Dp′) is the coagulation coefficient of particles of diameters Dp and Dp′, representing particles inside the size bin of *J*_Dp_ and those of pre-existing particles, respectively. *N*_Dp′_ is the number concentration of the pre-existing particles.

The condensation sink, CS, is the rate at which gaseous precursors are lost to pre-existing particles.4
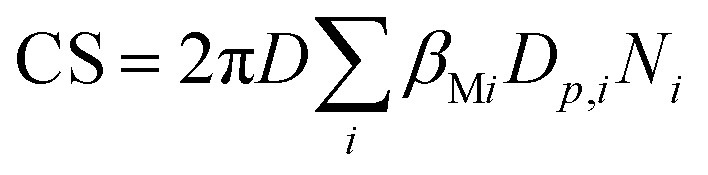


Here, Dp and *N* are the particle diameter and its corresponding number concentration, respectively. *β*_M_ is the transitional regime correction factor. *D* is the diffusion coefficient of precursor vapor, here H_2_SO_4_ and is calculated as per Fuller *et al.*:^53^5
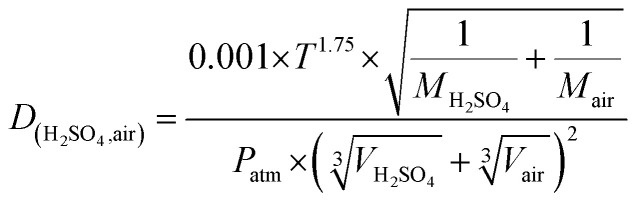


Here, *T* is the measured temperature, *M* is the molar mass, *P*_atm_ is the atmospheric pressure, and *V* is the diffusion volume.

#### Volatility basis set

For determining an approximate volatility of the individual organic products measured on site, we rely on a previous combination of semi-empirical methods, theoretical model calculations and parametrization to derive the volatility of oxygenated organic compounds measured at Qvidja. Similar to Stolzenburg *et al.*^54^ and Simon *et al.*,^40^ we use the two-dimensional volatility basis set (2D VBS) introduced by Donahue *et al.*,^55^ which uses the molecular composition of a molecule and its known volatility from parameterizing the saturation vapor pressure of an unknown molecule according to its mass and oxidation state.^56^ The estimated volatility of an individual molecule can be derived as proposed by Mohr *et al.*^57^ and adjusted by Stolzenburg *et al.*:^58^6
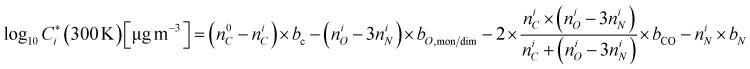


Here, *n*^*i*^_*C*_, *n*^*i*^_*O*_ and *n*^*i*^_*N*_ represent the number of carbons, oxygens and nitrogens in the organic molecule *i*, respectively. *n*^0^_*C*_= 25, *b*_C_ = 0.475, *b*_*CO*_ = −0.3 and *b*_*N*_ = 2.5, *b*_O,mon._ = 1.4 and *b*_O,dim._ = 1.17.

The volatility at ambient temperature, *T* (in Kelvins), can be calculated using the Clausius–Clapeyron equation:7
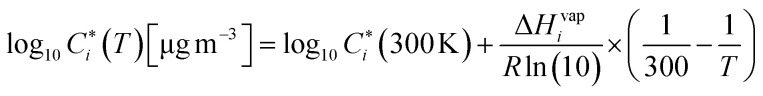
where 
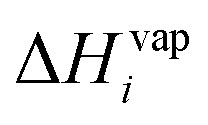
 is the evaporation enthalpy and can be approximated according to Donahue *et al.*^59^ as:8
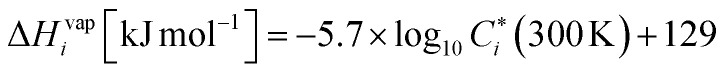


The volatility classes are distributed as follows:

SVOCs (Semi-Volatile Organic Compounds): 0.3 μg m^−3^ < C* (*T*) < 300 μg m^−3^.

LVOCs (Low Volatility Organic Compounds): 3 × 10^−5^ < C* (*T*) < 0.3 μg m^−3^.

ELVOCs (Extremely low volatility organic compounds): 3 × 10^−9^ < C* (*T*) < 3 × 10^−5^ μg m^−3^.

ULVOCs (Ultra-low volatility organic compounds): C* (*T*) ≤ 3 × 10^−9^ μg m^−3^.

In this study, due to their low observed concentrations, the concentration of ELVOCs also included the concentration of ULVOC, *i.e.*, ELVOCs: C* (*T*) ≤ 3 × 10^−5^ μg m^−3^.

## Results

### NPF at an agricultural land

At this measurement site, although located close to a forest, NPF events were rather unique. Some NPF days were characterized by a burst of particles, similar to a bump or ‘apple-type’ events indicative of local NPF events,^44,45^ and such bursts were observed starting from the smallest diameters, here 1.2 nm (Fig. 1). Interestingly, NPF events previously observed within agricultural lands at other locations did not show similar features^18,19^ and exhibited ‘banana-type’ size distributions. In the aforementioned studies, as the particle size distributions below 5 nm were not measured, due to instrument limitations, one could not rule out that these events are transported rather than locally formed due to agricultural emissions. In Qvidja, the clustering continued between 1 and 6 hours, and the formed clusters grew in diameter up to 20 nm at our measurement site. Hereafter, these ‘apple-type’ NPF events are referred to as local clustering events. Such clustering events are indicative of a nearby hot spot of NPF precursors, causing intense yet local formation and early growth of new aerosol particles, reported earlier in some coastal and polar environments.^39,60–62^ At Qvidja, local clustering constituted 33% of the measurement period (spring and autumn 2019) (Fig. S6†), with the highest frequency in May.

Local events in Qvidja were observed in both spring and autumn regardless of fertilization or harvesting (Fig. 1). On some days, local clustering events had limited growth where their end diameter did not exceed a few tens of nm. But on most of the days, local clustering events merge with regional events and together are observed to reach up to several hundreds in nm. Alternatively, regional events were observed on some days, and also during both spring and autumn, without the occurrence of local clustering (Fig. S4†). In Fig. S5,† NPF events in May are shown for both Qvidja and Hyytiälä. The frequency of events in each of the two locations in shown in Fig. S6.† Interestingly, although the sulfuric acid concentrations are similar in both Qvidja and Hyytiälä, see Fig. S7,† the intensity and the duration of the events vary substantially in both locations. Additionally, the starting diameter of the events in Qvidja is smaller, down to 1.2 nm, than those in Hyytiälä, leading to a similar conclusion to that described above, that those measured in Qvidja are rather local clustering events, compared to the events in Hyytiälä which are transported to the measurement location. We here note that ‘transported events’ are regional events arriving to the measurement location *via* vertical or horizontal transport.^45^ It is important to note that the ammonia concentrations in Hyytiälä (50–150 pptv)^63^ are several orders of magnitude lower than those measured in Qvidja (100–10 000 pptv). In the following sections, we investigate the vapors and factors promoting and inhibiting local clustering events in Qvidja.

### Precursor vapors driving local clustering and growth

**Fig. 2:**
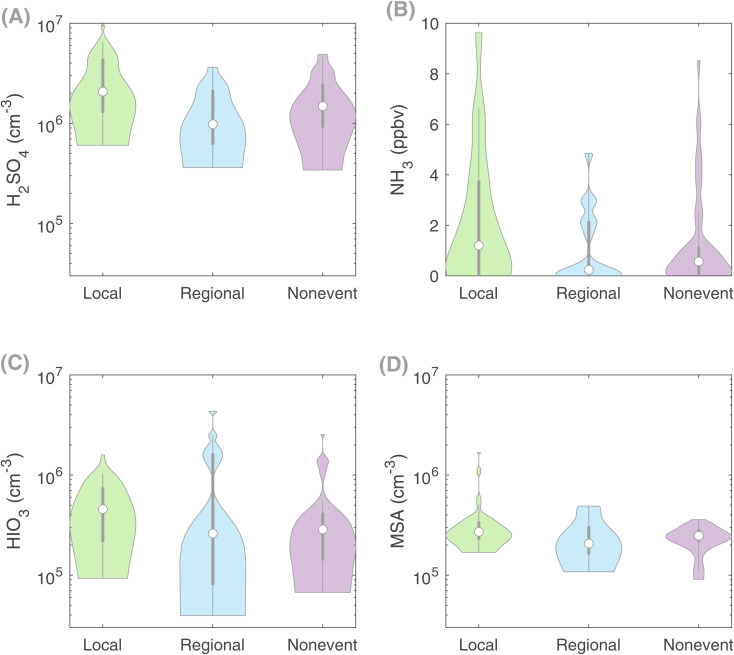
Violin distribution plots showing the availability of different precursor vapors: (A) sulfuric acid, H_2_SO_4_, (B) ammonia, NH_3_, (C) iodic acid, HIO_3_ and (D) methanesulfonic acid, MSA, on local clustering event days, regional new particle formation event days and non-event days. The data include spring (April and May) 7:00–16:00 LT. Violin plots are a combination of boxplots and a kernel distribution function on each side of the boxplots. The white circles define the median of the distribution and the lower and upper edges on the inner grey boxes refer to the 25th and 75th percentiles, respectively. Compared to both regional events and non-event days, the concentrations of H_2_SO_4_ and NH_3_ are substantially higher on local clustering event days, explaining the occurrence of clustering events.

Sulfuric acid, due to its low volatility, is found to be the main driver of clustering and NPF in many environments around the world,^64^ especially when stabilizing vapors such as ammonia and amines are readily available.^10^ This appears to be the case in our studied agricultural land. The daytime (7:00–16:00 LT) median concentration of H_2_SO_4_ during our measurement period in spring was 1 × 10^6^ cm^−3^ with a maximum of 1 × 10^7^ cm^−3^ (Fig. 2). This concentration level lies within the range of sulfuric acid concentrations observed in such rural and semi-urban environments,^65^ where H_2_SO_4_–NH_3_, in the presence of organic vapors, explains the NPF rates.^66–68^ On days with local clustering observed at Qvidja, the median H_2_SO_4_ concentration (2.1 × 10^6^ cm^−3^) was more than 2 times higher than that observed on days with only regional NPF ([H_2_SO_4_]_Reg_ = 9.8 × 10^5^ cm^−3^) and 1.4 times higher than that on non-event days ([H_2_SO_4_]_NE_ = 1.5 × 10^6^ cm^−3^) (Fig. 2). This higher H_2_SO_4_ concentration can be attributed to a higher brightness parameter or clear-sky conditions favoring clustering,^49^ while the effect of the condensation sink (CS) is expected to be minute given the similarly low CS value on both cluster event and non-event days (Fig. S8†). A similar conclusion has been drawn from other studies over agricultural lands, where the CS does not appear to be the sole factor responsible for explaining the absence of NPF on some days.^18–20^

Additionally, the median NH_3_ concentration was approximately one order of magnitude higher on days with local clustering (1.21 ppbv) compared with days when no such clustering is observed ([NH_3_]_Reg_ = 0.23 ppbv and [NH_3_]_NE_ = 0.57) (Fig. 2). These significantly lower NH_3_ concentrations, together with lower H_2_SO_4_ concentrations, could explain the overall absence of clustering on regional NPF and non-event days. At Qvidja, ammonia is released from the horse stable and cow farm. Fertilization occurred on two instances throughout our measurement period, affecting the NH_3_ concentration without showing an immediate effect on the clustering frequency or intensity (Fig. S9†), which signifies that NH_3_ might not be the determining factor of particle formation intensity.

**Fig. 3:**
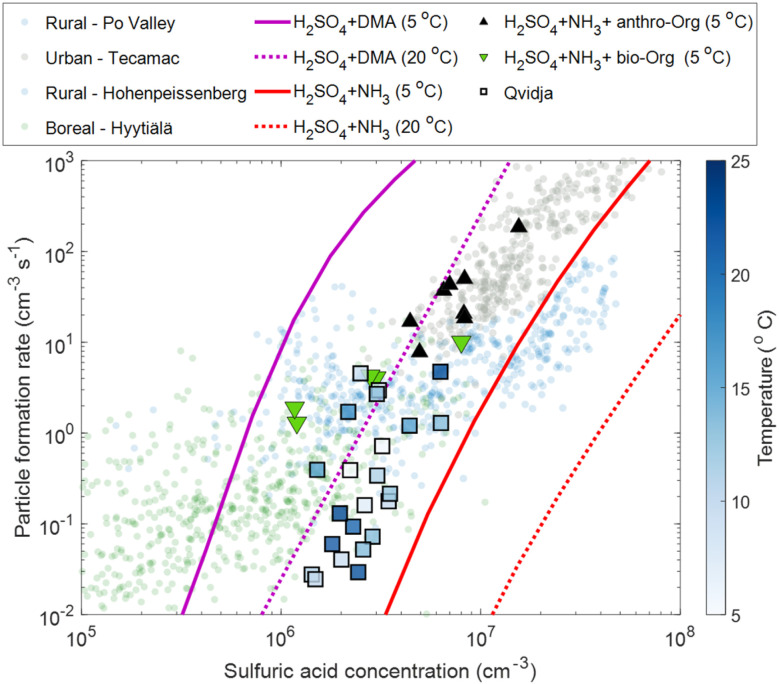
Particle formation rate (*J*_1.5_) as a function of H_2_SO_4_ concentration, daily averages (12:00–16:00 LT) – squares, colored with ambient temperature (average temperature is 13 °C). Parametrization based on chamber measurements of H_2_SO_4_ + NH_3_ (2 ppbv) ammonia at 5 °C (solid red line) and 20 °C (dotted red line) is shown.^70^ Similarly, parametrization based on chamber measurements of H_2_SO_4_ + dimethylamine (DMA – 4pptv) at 5 °C (solid magenta line) and 20 °C (dotted magenta line) is shown.^70^ Green triangles are H_2_SO_4_ + NH_3_ (0.1–1 ppbv) at 5 °C in the presence of constant monoterpenes (α-pinene and Δ-3-carene) and NO_*x*_ from Lehtipalo *et al.*^67^ Black triangles are H_2_SO_4_ + NH_3_ (1–2 ppbv) in the presence of anthropogenic organics.^70^ Filled translucent points in the background are atmospheric measurements from the boreal forest (green), rural locations^90,91^ (pink) and polluted environments (gray).^92^ The measured particle formation rates from Qvidja are at 1.5 nm, while those from chamber measurements are measured at 1.7 nm ^51^ and extrapolated to 1.5 nm using the Kerminen and Kulmala equation.^93^ The particle formation rates from atmospheric observations are also reported at 1.5 nm.

**Fig. 4:**
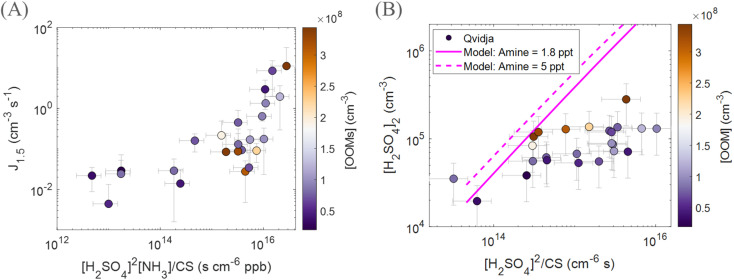
Mechanism of new particle formation at Qvidja. (A) Correlation between the particle formation rate at 1.5 nm and precursor concentrations [H_2_SO_4_]^2^ × NH_3_/CS representing the first cluster made of H_2_SO_4_ and NH_3_ in spring in Qvidja, daily averages (12:00–16:00 LT). (B) Measured sulfuric acid dimer as a function of its theoretically calculated concentration, daily averages (12:00–16:00 LT). Pink lines are theoretical expectation of sulfuric acid dimer in the presence of 1.8 pptv amines (solid line) and 5 pptv (dashed line), described in Cai *et al.*,^71^ and markers are colored by oxygenated organic molecule concentration (OOM).

In order to understand the mechanism behind particle formation in Qvidja, we first quantify the intensity of NPF, which can be described by the rate at which newly formed particles enter a certain size range, in our case 1.5–3 nm (Fig. S10†), relevant for clustering. Although the median formation rate of 1.5 nm particles, *J*_1.5_, was 0.15 cm^−3^ s^−1^ at Qvidja, *J*_1.5_ reached hourly average values as high as 70 cm^−3^ s^−1^ (*e.g.*, April 20, 2019 and May 22, 2019) – much higher than the formation rates observed in boreal forest environments and comparable to those observed in urban environments.^69^ In Fig. 3, we plot the particle formation rate *J*_1.5_ as a function of H_2_SO_4_ monomer concentration. Although we observe higher particle formation rates at higher sulfuric acid concentration, the data points show quite a spread indicating the possibility of multiple formation pathways or the involvement of additional vapors besides H_2_SO_4_. In addition, while the temperature does not seem to affect the relationship between sulfuric acid concentration and the particle formation rate, it influences the concentrations of OOMs and therefore the capability of the particles to grow to larger diameters. For instance, if we focus on the data points (Fig. 3) with average temperatures close to 5 °C, we find that these points fall far above the ternary sulfuric acid–NH_3_ nucleation line at 5 °C but below the ternary sulfuric acid–DMA nucleation line at 5 °C. This observation draws two conclusions, (1) sulfuric acid–NH_3_ alone cannot explain the observed nucleation rates, and (2) the role of DMA is not evident (*e.g.*, too low concentration of DMA in Qvidja). Nevertheless, the data points from Qvidja fall on the same line of data points obtained from chamber experiments where H_2_SO_4_, NH_3_ and OOMs were involved in the particle formation.^67,70^ Such an observation suggests the role of OOMs in supporting the sulfuric acid–NH_3_ nucleation. A similar conclusion can be drawn also for the data points from Qvidja at higher temperatures, where sulfuric acid–NH_3_ alone cannot explain the nucleation rates, but the points still fall below the sulfuric acid–DMA line, which confirms that the nucleation is weaker than sulfuric acid–DMA, at least at 4 pptv. When compared to other measurement locations, Qvidja seems to fall at the interface between the rural and the boreal forest environments. This does not come as a surprise given the nature of Qvidja's location which is an agricultural land surrounded by boreal forests. Such an observation could point towards a synergistic role of the agricultural land and forest in particle formation, *i.e.*, H_2_SO_4_, NH_3_ and OOMs were involved in the particle formation. In Fig. 4A we plot *J*_1.5_ as a function of [H_2_SO_4_]^2^ × [NH_3_]/CS representing the first cluster made of H_2_SO_4_ and NH_3_. A positive correlation is seen especially for the data points with *J*_1.5_ exceeding 0.1 cm^−3^ s^−1^ while the *J*_1.5_ does not show any apparent dependence on the OOM concentration. Previous studies have shown that particle formation from OOMs is slow compared to H_2_SO_4_ and NH_3_, while the addition of OOMs to the system of H_2_SO_4_ and NH_3_ enhances the particle formation rate by strengthening the attachment between the molecules in this size range.^67^ Some of the data points of Qvidja fall close to the parametrized H_2_SO_4_ + dimethylamine line (Fig. 3), which could signify a contribution of amines in particle formation, yet direct amine measurements at the location were not performed given the instrument limitations. In order to rule out the contribution of amines, we calculated the theoretical steady-state H_2_SO_4_ dimer concentration based on a 1.8 pptv amine (and 5 pptv) concentration following the method presented by Cai *et al.*^71^ and compared it to the measured dimer concentration (Fig. 4B). Most of our measurements fall below the 1.8 pptv (and 5 pptv) amine line, providing evidence that H_2_SO_4_ in Qvidja is not clustered to amines, making NH_3_ (present in substantially high concentrations of up to 10 ppbv) the most plausible base stabilizing H_2_SO_4_. Only 8% of the data points fall on or above the amine line during these days, so the amine contribution to nucleation cannot be ruled out completely. However, data points that fall closer to the amine line are marked by higher OOM concentrations, which suggests a potential role of organics in fixing H_2_SO_4_. It is worth mentioning here that while observations of gas phase amines over agricultural lands remain missing, measurements of very high concentrations of amines from livestock discharge have been reported.^72^ Kürten *et al.*^73^ estimated a concentration of different amines to range between 1 and 5 pptv in a rural area in Germany located around 1 km from a dairy farm and dairy producing factory. Interestingly, in the boreal forest environment in Hyytiälä, the concentrations of DMA measured during spring were in a similar range between the detection limit and 4.1 pptv but were attributed to biogenic sources.^74^ Although higher concentrations of amines would be expected in urban areas given the abundance of industry and traffic, the concentrations of amines in Beijing^71^ and other urban locations were within the same range as observed in rural and semirural locations, see Hemmilä *et al.*^74^ and references therein. Therefore, we expect the DMA concentration in Qvidja to fall in the same range as the aforementioned studies (1–5 pptv).

Finally, to eliminate the possibility of the contribution of other clustering mechanisms, given the proximity of the Qvidja farm to the coast (Fig. S1†), we examined the concentrations of iodic acid (HIO_3_) and methane sulfonic acid (MSA). Although the concentrations of both acids were higher on days when clustering was observed than on the other days (Fig. 2), the overall concentrations of these acids (median HIO_3_ = 4.6 × 10^5^ cm^−3^ and median MSA = 2.7 × 10^5^ cm^−3^) were too low to explain the observed cluster formation rates based on our current understanding.^39^ In addition, we found no correlation between the particle formation rate *J*_1.5_ and either acids, confirming that they do not participate in particle formation in Qvidja.

Altogether, the results suggest a synergistic role of H_2_SO_4_, NH_3_ and OOMs in particle formation in Qvidja, and therefore, a synergy between regional emissions (SO_2_ and thus H_2_SO_4_), soil emissions (NH_3_) and plants (OOMs) is needed to form particles and grow them to climate relevant sizes (discussed in the next sections).

### Why do we not observe clustering on every sunny day?

**Fig. 5:**
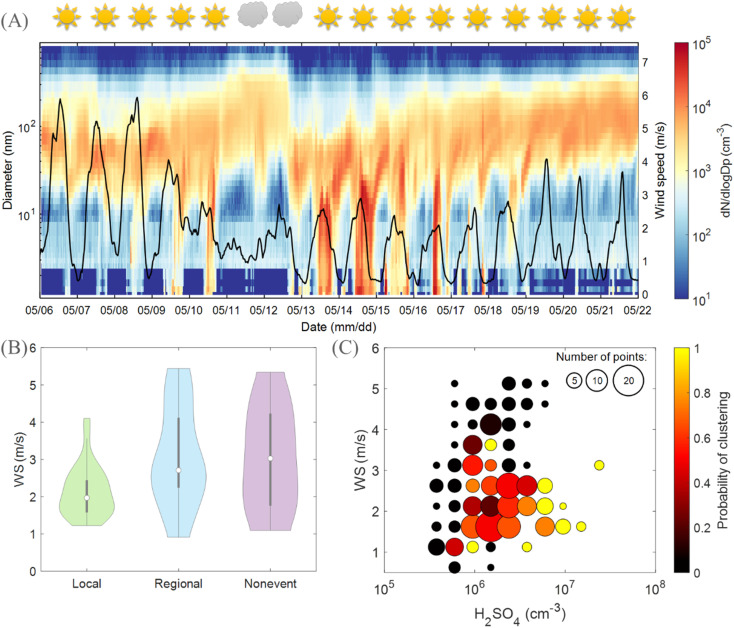
Role of meteorology in clustering in Qvidja. (A) Particle number size distribution of consecutive clustering events in Qvidja. The sun and clouds on the upper *x*-axis refer to whether each of these days is classified as a sunny or cloudy day based on the median brightness parameter between 9:00 and 12:00. If *P* > 0.7, the day is classified as sunny, and hence a sun is displayed. A time series of the wind speed is shown as the solid black line, on the right axis. (B) Violin distribution plots showing the wind speed in spring 2019 (7:00–16:00 LT) on cluster event days, regional new particle formation event days and non-event days. Violin plots are a combination of boxplots and a kernel distribution function on each side of the boxplots. The white circles define the median of the distribution and the lower and upper edges on the inner grey boxes refer to the 25th and 75th percentiles, respectively. (C) Clustering event probability distribution based on the wind speed (WS) and H_2_SO_4_ concentrations. Marker size indicates the number of days included in the probability calculation within every cell. The highest probability of a clustering event is on the bottom right corner of the figure combining a low wind speed and high H_2_SO_4_ concentration. The figure shows the necessity of sufficient solar radiation (clear sky conditions), and hence sufficient H_2_SO_4_ concentration (>1 × 10^6^ cm^−3^) as well as a low wind speed preventing dilution of locally emitted precursors (here NH_3_, see Fig. 1 and 2) for the occurrence of clustering events in Qvidja. Note that fertilizers were added to the field on May 8th which disturbed the measurements and the day is excluded from further analysis.

Although it is confirmed that clustering events at Qvidja were driven by H_2_SO_4_ which was abundant on clear-sky days, we did not observe an event on every sunny day (Fig. 5A). This observation endorses that there must be another factor playing a role in determining the occurrence of local clustering events at Qvidja. Here, the wind speed (WS) appears to play a crucial role. When the WS exceeded 3.5 m s^−1^, no local clustering was observed. This is consistent with Fig. 5B, which shows a clear divergence of the WS between days when a local clustering event is observed and when not. A high WS plausibly dilutes precursor vapors needed for clustering. Fig. 5C shows further that the probability of having a clustering event is highest in the lower right corner which corresponds to sufficient H_2_SO_4_ and low WS. In other words, only when sufficient H_2_SO_4_ is available, a low wind speed ensures stagnation and thus the availability of locally emitted NH_3_ and organic compounds permitting the participation of the agricultural land in the cluster stabilization and growth process.

### Role of organics in particle formation and growth in Qvidja

The role of organics in forming and growing particles in an atmospheric setting remains challenging given the multiple emitted vapors and various oxidation mechanisms resulting in thousands if not millions of compounds floating in the air. However, organic molecules have been shown to grow the freshly formed particles, and the lower the volatility of the available vapor the higher its contribution is in growing particles of smaller sizes.^53,57^

In Qvidja, the particle growth rates were remarkably high, and in some cases too high to be quantified (Fig. 1). What is most interesting is that the GR does not drop substantially with increasing diameter.^75^ For instance, the GR on May 13th (1st peak – Fig. 1B) is estimated to be 6.3 nm h^−1^ for the sub-3 nm particles and 5.7 nm h^−1^ for the particles between 3 and 7 nm. Meanwhile the GR on May 16th (Fig. 1C) is estimated to exceed 9.3 nm h^−1^ for the sub-3 nm particles and 9.6 nm h^−1^ for the 3–7 nm particles. We note that the GR calculations are subject to high uncertainties for Qvidja, given the extremely high GR compared to other locations globally.^69,76^ Such a GR cannot be explained by the available H_2_SO_4_ concentrations, which highlights the importance of organics for particle growth. We note that the growth rate in the sub 3 nm size range has not been previously reported for any agricultural land, and therefore we cannot directly compare our observations to other locations. However, the GR of particles exceeding 5 nm measured in a French agricultural land is comparable to our observations;^19^ however, the authors of the aforementioned study could not rule out the contribution of anthropogenic emission from the Paris city center ∼25 km from the measurement location. This makes our study location unique as it is subject to mainly biogenic emissions from plants and surrounding forests.

**Fig. 6:**
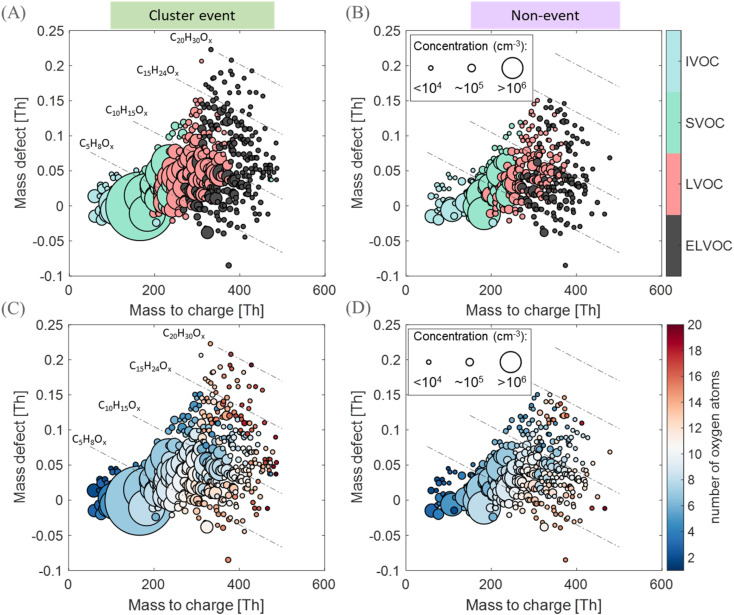
Chemical composition and volatility distribution of molecules in Qvidja. Mass defect plots showing the volatility distribution of molecules measured on a (A) local clustering event day on May 15th, 2019 and on a (B) non-event day on May 6th, 2019. Mass defect plots showing the number of oxygen atoms per molecule measured on a (C) local clustering event day on May 15th, 2019 and on a (D) non-event day on May 6th, 2019. A mass defect plot is composed of plotting the mass defect (difference between the exact mass and integer mass) *versus m*/*z* of gas-phase OOMs measured with the nitrate anion CI-APi-TOF. For clarity, only signals of organics are displayed in the plot. Each circle represents a particular molecular composition. The size of the marker is proportional to the concentration of each molecule measured by the nitrate CI-APi-TOF.

In Fig. 6 and S11,† we display mass defect plots and the volatility distribution set from a local clustering event day and a non-event day. A comparison between the volatility distribution of molecules as well as their carbon and oxygen content is also shown. Most of the molecules appear to contain 5 (isoprene-backbone) or 10 (monoterpene-backbone) carbon atoms; although given the surrounding boreal forest environment, one would expect a dominance of monoterpenes over isoprene.^77^ However, croplands are expected to emit isoprene,^78^ leading to a higher isoprene to monoterpene concentration ratios compared with the boreal forest environment.^79^ During both the event and non-event days, the distribution of the number of carbons remains similar, and thus no variation in the emission type is expected. Additionally, we do not observe a clear enhancement of OOM concentrations on local event days compared to non-events, as the former are mostly affected by temperature and GPP (Fig. S10†). However, a higher degree of oxygenation represented by higher oxygen numbers and a shift towards a lower volatility is observed on the clustering event day, compared with a non-event day (Fig. 6). Such an observation could be related to a higher photo-oxidation of available vapors, as the brightness parameter on the cluster event day (*P*_9:00–18:00_ = 0.95) is a factor of 1.2 higher than on the non-event day (*P*_9:00–18:00_ = 0.78), and the CS is similar in both cases (CS_9:00–18:00_ = 0.003 s^−1^). Another explanation could be related to the wind speed. The WS is a factor of 2.7 lower of the cluster event (WS_9:00–18:00_ = 1.9 m s^−1^) day compared to the non-event day (WS_9:00–18:00_ = 5.7 m s^−1^). Although OOMs are of regional scale, a slower wind speed allows for stagnation and is expected to accumulate vapors^80^ facilitating BVOC oxidation by increasing the production rate of oxidation products as more BVOCs are expected in a smaller volume mixed layer.

**Fig. 7:**
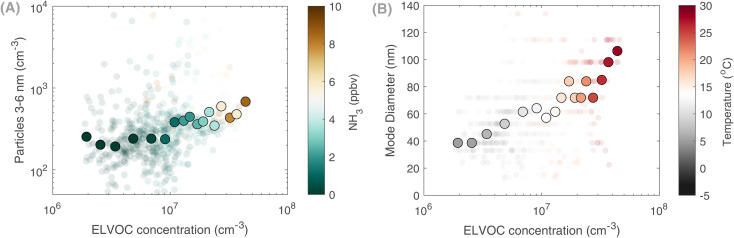
Contribution of low volatility organics to particle growth. (A) Particle number concentration in the size bin 3–6 nm as a function of extremely low volatility organic compounds, color-coded with ammonia concentration during May 2019 and (B) particle mode diameter as a function of extremely low volatility organic compounds, color-coded with temperature during May 2019. In both (A) and (B) the data are binned to the ELVOC concentrations, and the hourly averages are shown in the background.

Moreover, the relationship between particle growth rates and OOMs remains challenging given the measurement limitations of both the fast growth rates and some of the OOMs. However, we do observe during May a positive correlation between extremely low volatility (ELVOC) OOMs and the particle number concentrations of the growing mode (3–6 nm), especially at higher NH_3_ concentrations (Fig. 7A). This signifies the synergistic role of the agricultural land (NH_3_) and vegetation (organic vapors) in particle formation and growth. Besides, the role of organics on a regional scale is highlighted in Fig. 7B, where a correlation between ELVOCs and the mode diameter of the growing particles is shown. At higher temperatures, higher concentrations of ELVOCs allow for particles to grow to larger sizes, amplifying their climatic effects. In Qvidja, the observed OOM concentrations cannot explain the measured growth rates, *e.g.*, similar to what has been observed in Hyytiälä^57^ or from chamber experiments;^53^ yet, the concentration of ELVOCs was significantly higher on days when particles exceeded 40 nm in diameter. Such an observation suggests a vital role of condensable OOMs in particle growth, even on a regional scale.

### Climatic importance of aerosol particle production associated with agricultural lands

In order to assess the climatic importance of agricultural lands *via* their production of new aerosol particles, we compare the general character of the particle formation process observed in Qvidja to that observed at the well-studied SMEAR (Station for Measuring Ecosystem–Atmosphere Relations) II station, located about 200 km from Qvidja inside a boreal forest environment in Hyytiälä (Fig. S1†). Boreal forests, by producing new aerosol particles and making them to grow in size, have been shown to be a large source of cloud condensation nuclei (CCN) ranging from regional to even larger scales,^81–83^ and this CCN production causes a cooling effect on climate *via* aerosol–cloud interactions.^82,84–86^

**Fig. 8:**
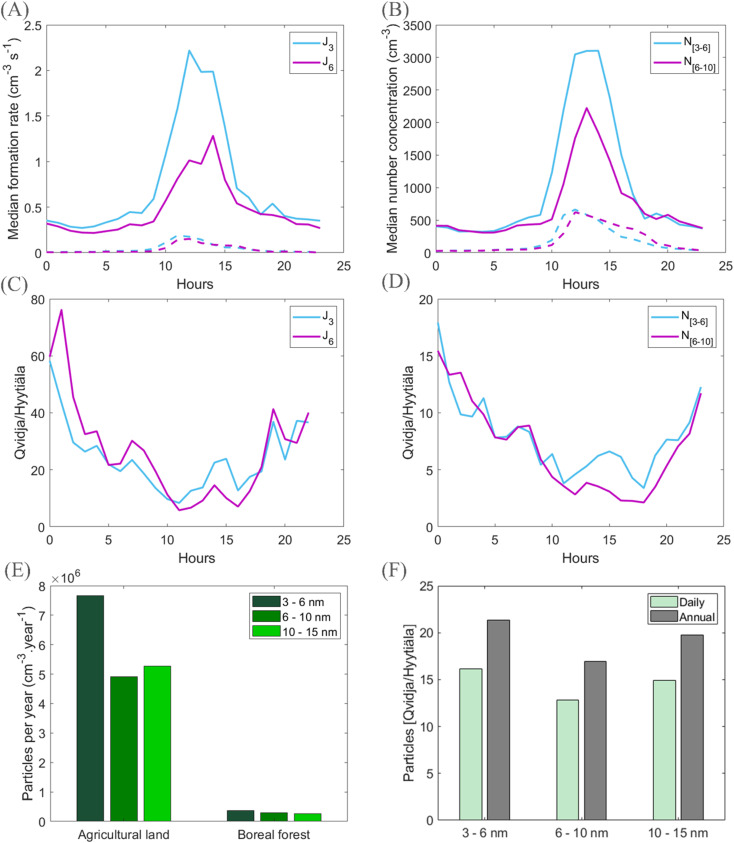
New particle formation at an agricultural land and in the boreal forest. (A) Particle formation rates during event days in Qvidja (solid lines) and in Hyytiälä (dashed lines) at different sizes. (B) Number concentration of particle ‘event’ days in Qvidja (solid lines) and in Hyytiälä (dashed lines) at different sizes (*J*_3_ and *J*_6_ refer to particle formation rates at 3 and 6 nm, respectively). (C) Ratios of formation rates at different sizes. (D) Ratios of number concentrations at different sizes. (E) The annual production of particles in different size ranges in Qvidja and in Hyytiälä. (F) the ratio of daily and annual production of particles in different size ranges at Qvidja to Hyytiälä. The grassland produces up to 15 times more particles per cm^3^ per day and more than 20 times more particles per cm^3^ per year in the size ranges between 3 and 10 nm. These particles have shown to be capable to grow to climate relevant diameters and to contribute to regional NPF and cloud condensation nuclei budgets.

Fig. 8 compares the diurnal behavior of particle formation rates and resulting particle number concentrations between Qvidja and Hyytiälä during days with clustering and NPF days, as well as both daily and annual budgets of the produced particles in different size ranges. We may see substantially higher formation rates of both 3 and 6 nm particles in Qvidja (Fig. 8A), both exceeding the corresponding formation rates in Hyytiälä by a factor between about 10 and 20 during the day-time with the most intense NPF (Fig. 8C). The number concentrations of 3–6 nm and 6–10 nm particles reach median values of about 3000 and 2000 cm^−3^, respectively, in Qvidja, while the corresponding values are both about 500 cm^−3^ in Hyytiälä (Fig. 8B). Over the course of the day, number concentrations of 3–6 nm and 6–10 nm particles observed in Qvidja exceed those in Hyytiälä between factors of about 3 and 15 (Fig. 8D). On a daily and an annual basis, and when taking into account the frequency of clustering in Qvidja (33%), these processes produce between about 10 and 20 times more particles compared with NPF events in Hyytiälä (frequency ∼25%) (Fig. 8E and F, S6†), and this enhancement can be observed up to the size range of 10–15 nm.

In a boreal forest environment, the growth of newly formed particles up to CCN sizes has been observed to take place over time scales of roughly 1 to 3 days of air mass transport.^83,85^ Although it remains impossible to directly measure CCN production associated with clustering events observed at Qvidja, there are several indirect lines of evidence to suggest that a big fraction of the particles formed at Qvidja will eventually reach CCN sizes downwind this site. First, the observed particle growth rates between boreal forests and other rural environments tend to be relatively similar.^7,76^ This and the fact that Qvidja is surrounded mainly by forests at larger scales over the land area indicate that the estimated CCN growth time scale of 1–3 days for boreal forests also applies for particles produced at Qvidja. Second, the main atmospheric sink of newly formed aerosol particles is usually their coagulation scavenging by larger, pre-existing aerosol particles.^87,88^ The strength of this sink decreases considerably in moderately polluted environments as the growing particles reach sizes larger than about 10–20 nm. Third, typical overall lifetime of sub-CCN size aerosol particles in the planetary boundary layer are about 1–2 days,^89^*i.e.*, comparable to what it takes for newly formed particle to grow into CCN sizes.

## Conclusions

Previous studies at Qvidja have shown the capability of agricultural grassland with improved farming practices to act as a carbon sink.^15^ This, together with the results of our study, shows that such farming practices and agricultural grasslands as a whole have the capacity to act as an effective neutralizer for a changing climate. The local clustering in agricultural fields contributes to regional NPF per square meter of land *ca.* 15–20 times more than a corresponding area of boreal forests (Fig. 8). We found that locally emitted ammonia stabilizes regionally available sulfuric acid, leading to NPF events with intensities *ca.* 15–20 times higher than those measured in neighboring boreal forest environments. Clear sky conditions ensure sufficient H_2_SO_4_ concentrations (>1 × 10^6^ cm^−3^), while a low wind speed (<3.5 m s^−1^) ensures the contribution of NH_3_ emitted from the grassland. Once aerosol particles are formed, low volatility organic compounds, here ELVOC, contribute to the growth of particles to CCN and accumulation mode sizes, so that they can influence the radiative forcing balance. Such observations provide insights into the possibility of agricultural fields to have pro-environmental characteristics, as opposed to previous understanding of being solely greenhouse gas emitters and thereby climate harming.

During the measurement campaign, we observed unique ‘local clustering’ NPF events which do not resemble the regional NPF events observed over the past 25 years in the boreal forest in Finland. Although sufficient sulfuric acid is needed to initiate NPF in Qvidja, the local clustering events do not occur at a specific time of the day, in comparison to other locations where NPF events tend to start concurrent with sunrise when the concentrations of low volatility gaseous precursors increase most rapidly due to active photochemistry. Local clustering with regional NPF events (‘banana-like’ events) are also observed in Qvidja. Our aim is to understand the drivers behind these different types of NPF events, their precursor sources, and the chemistry behind them.

If generally applicable, the above analysis indicates that compared with boreal forests alone, agricultural land areas are 10–20 times more efficient in producing growing aerosol particles and probably several times more efficient in eventually producing new CCN. Globally, agriculture occupies more than 37% of Earth's land area while forests contribute to 30.7%.^17^ This situation is expected to change by 2050 with a retreat of agriculture land use in Europe.^16^ NPF events are found to contribute substantially to the global particle number concentration budget as well as affecting human health and furthermore to cloud condensation nuclei concentrations. Therefore, our observation of 15 times higher contribution to NPF of agricultural land, compared to boreal forests, is yet unaccounted for in global models; including it as a source for the global tropospheric particle number budget is a priority for determining and quantifying the potential for cooling presented by agriculture.

## Author contributions

Conceptualization of the study: L. D., V.-M. K., and M. K. Measurements: M. O. K., J. S., M. O. L., Y. W., L. H., J. D., and M. L. Data analyses: L. D., M. O. K., J. S., and M. O. L. Results interpretation: L. D., J. S., M. D., V.-M. K., T. P., and M. K. Writing–original draft: L. D. Writing–review & editing: V.-M. K. and M. K. Commenting: all.

## Conflicts of interest

There are no conflicts to declare.

## Supplementary Material

EA-003-D3EA00065F-s001
