# Supplementary material for: The synergistic role of sulfuric acid, ammonia and organics in particle formation over an agricultural land
Source: Environ Sci Atmos. 2023 Jul 7;3(8):1195–211. doi: 10.1039/d3ea00065f (PMC10413442; doi:10.1039/d3ea00065f)
Supplement: EA-003-D3EA00065F-s001 [file EA-003-D3EA00065F-s001.pdf]

Electronic Supplementary Material (ESI) for Environmental Science: Atmospheres.
This journal is © The Royal Society of Chemistry 2023




# Supplementary

## Synergistic role of sulfuric acid, ammonia and organics in particle formation over an agricultural land

Received 00th January 20xx,
Accepted 00th January 20xx

DOI: 10.1039/x0xx00000x

Lubna Dada[a,b,*], Magdalena Okuljar[a,β], Jiali Shen[a,β], Miska Olin[c], Yusheng Wu[a], Laura Heimsch[d], Ilkka Herlin[e], Saara Kankaanrinta[e], Markus Lampimäki[a], Joni Kalliokoski[c], Rima Baalbaki[a], Annalea Lohila[d], Tuukka Petäjä[a], Miikka Dal Maso[c], Jonathan Duplissy[a,f], Veli-Matti Kerminen[a], Markku Kulmala[a,*]

## This document contains one supplementary table and 11 supplementary figures.

Please do not adjust margins



*Table S1. Management of the agricultural land site at Qvidja farm in 2019.*

| Date | Management | Output | Input | in C |
|---|---|---|---|---|
| | | (kg DW $ha^{-1}$) | (kg $ha^{-1}$) | (g $m^{-2}$) |
| **8th of May 2019** | Fertilization | | -4606 | -43 |
| **11th of Jun 2019** | Harvest | 3107 | | 130 |
| **26th of Jun 2019** | Fertilization (mineral) | | | |
| **20th of Aug 2019** | Harvest | 1029 | | 43 |

 This journal is © The Royal Society of Chemistry 20xx

Please do not adjust margins

Please do not adjust margins



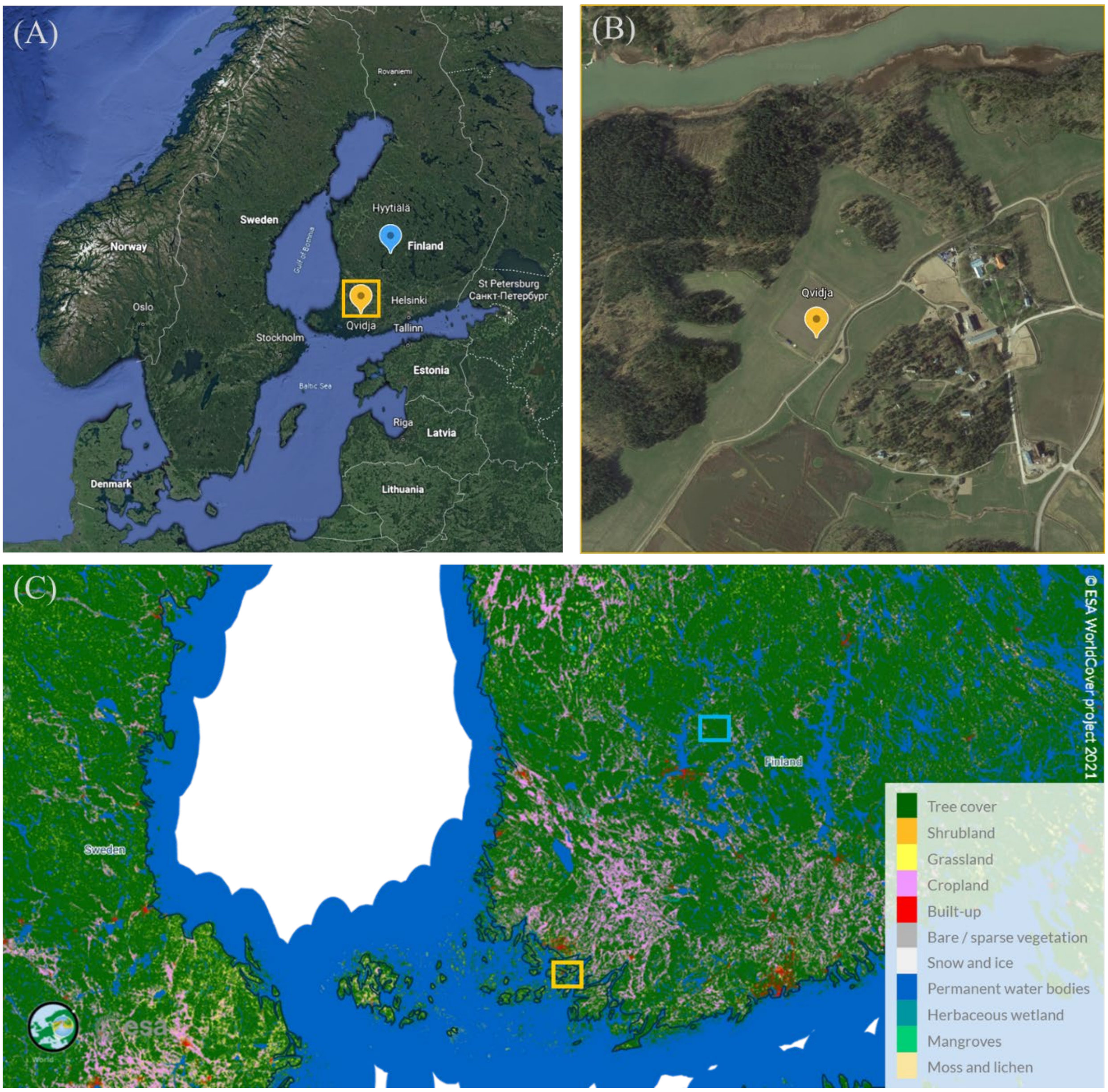


*Figure S 1 Location and land use information about Qvidja (agricultural land) and Hyytiälä (boreal forest), marked with yellow and blue respectively. Maps in (A) and (B) are extracted from Google Maps©, the land use cover in map (C) are extracted from ESA WorldCover ©.*



Please do not adjust margins

Please do not adjust margins



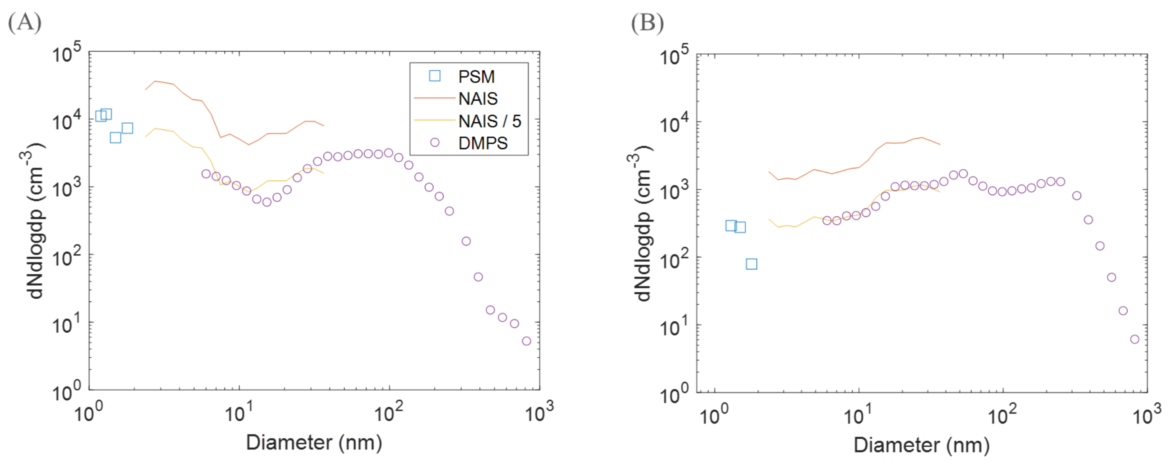


*Figure S 2 Example averaged particle number size distribution measured during particle formation event on May 16 and nonevent on May 23.*

 This journal is © The Royal Society of Chemistry 20xx

Please do not adjust margins

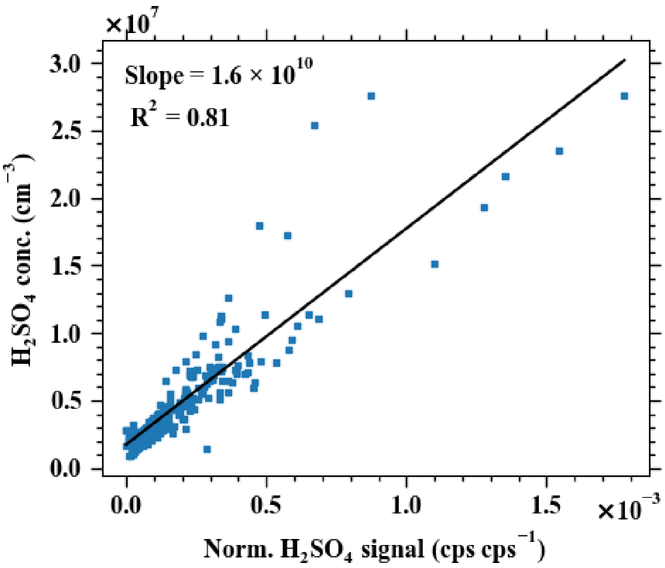


*Figure S 3 Linear fit between $NO_3^-$-CIMS (y-axis) and $NO_3^-$-MION-CIMS (x-axis) for the overlap time (13-23 June 2019) shows that the $H_2SO_4$ calibration coefficient in $NO_3^-$-MION-CIMS is around three times larger than that of $NO_3^-$-CIMS.*

Please do not adjust margins



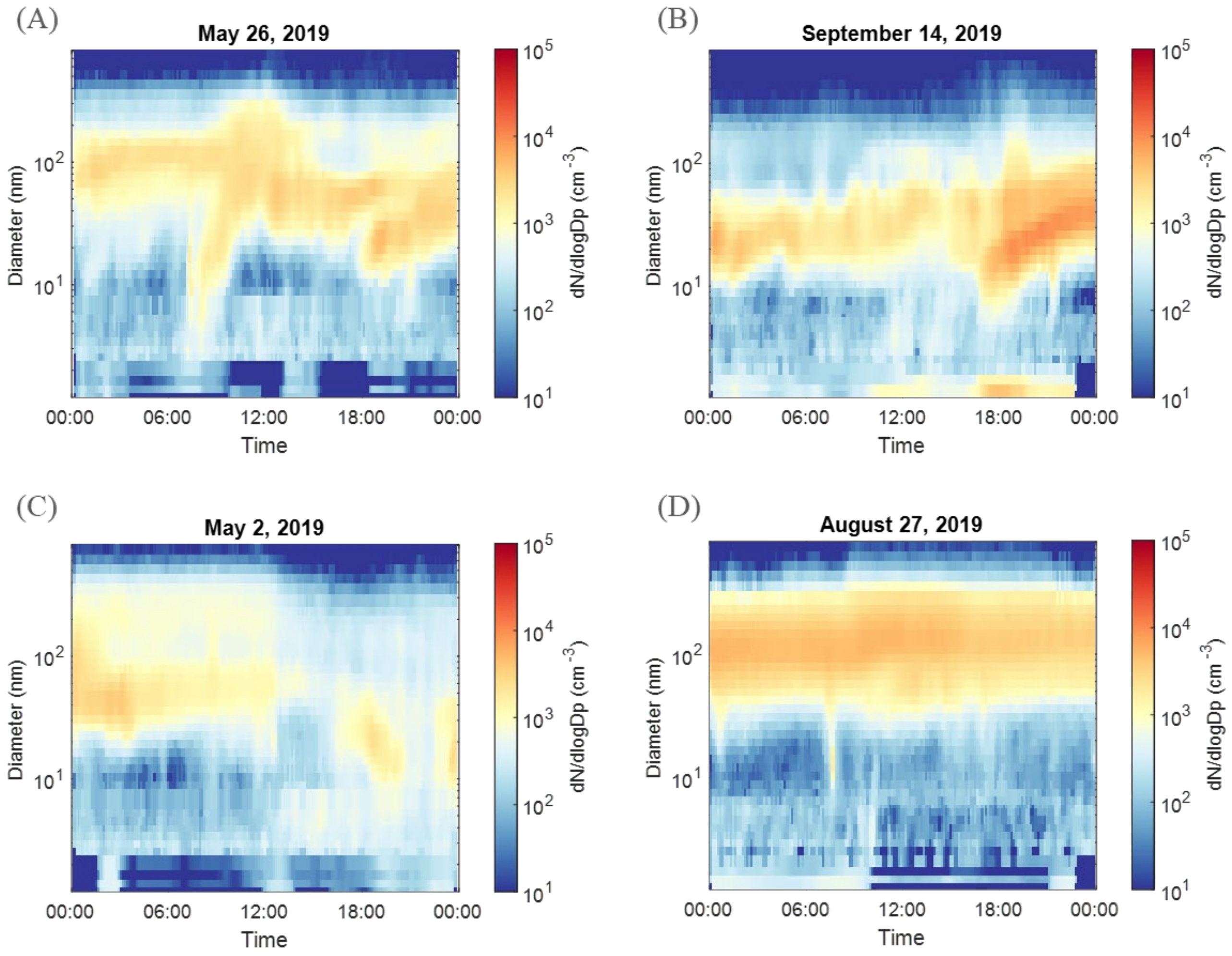


*Figure S 4 Examples of regional NPF events during (A) spring and (B) autumn. Examples of non-events during (C) spring and (D) autumn.*


This journal is © The Royal Society of Chemistry 20xx

Please do not adjust margins

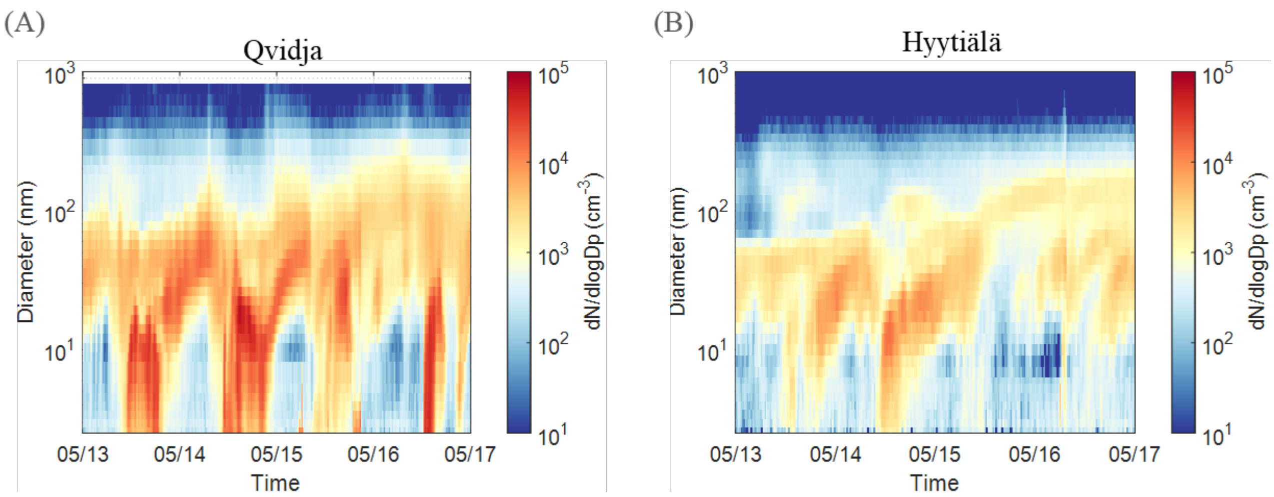


*Figure S 5 Particle number size distribution during simultaneous intense NPF events in Qvidja and Hyytiälä in May 2019. Comparison shows the higher intensity and smaller starting diameter of the events in Qvidja compared to Hyytiälä.*

Please do not adjust margins



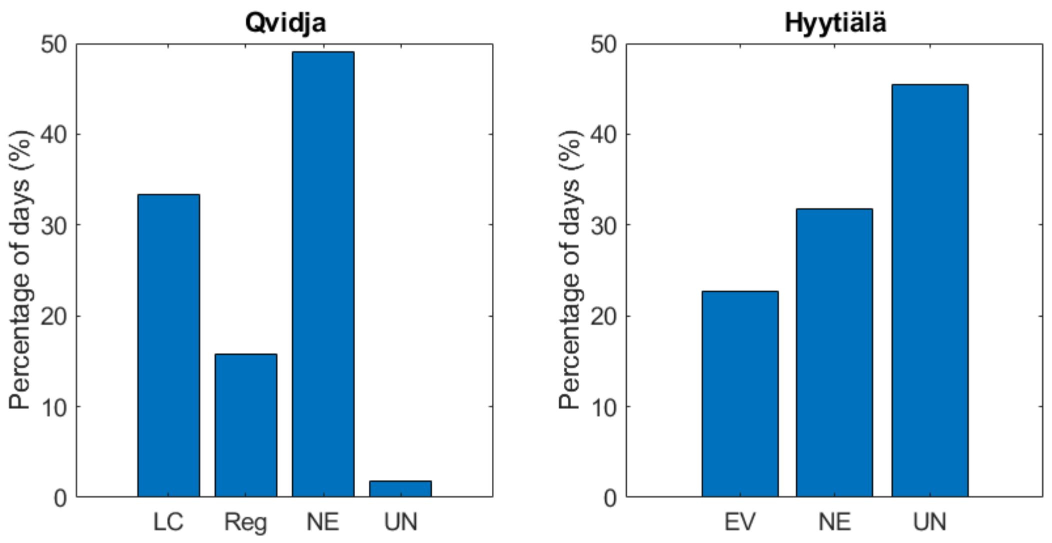


*Figure S 6 New particle formation event frequency at Qvidja and Hyytiälä. The acronyms LC, Reg, NE, UN, and EV stand for local clustering, regional NPF, non-event, undefined and event days, respectively. See methods section 3.1 for details on classification. Data from both sites includes same dates: spring and autumn 2019.*


This journal is © The Royal Society of Chemistry 20xx

Please do not adjust margins

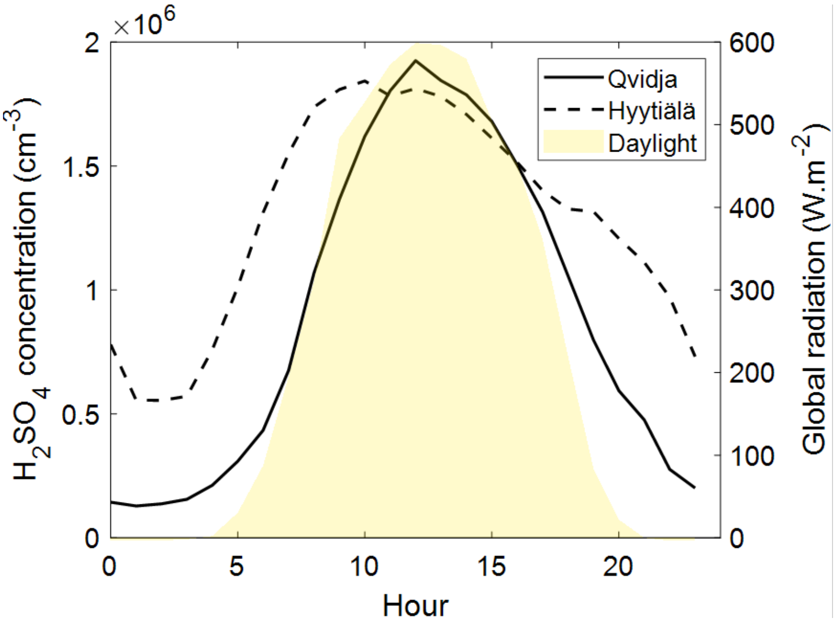


*Figure S 7 Diurnal sulfuric acid concentration in Qvidja (solid) and Hyytiälä (dashed) in May 2019. The shaded area represents daytime hours.*

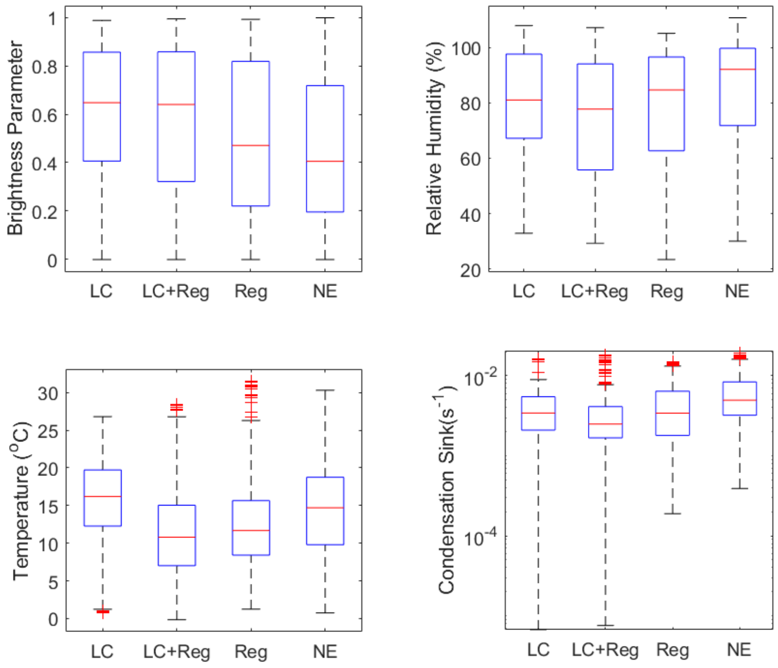


*Figure S 8 Brightness parameter, relative humidity, temperature and condensation sink on different days. The acronyms LC, Reg, and NE stand for local clustering, regional NPF and non-event. The red line represents the median of the data and the lower and upper edges of the box represent the 25th and 75th percentiles of the data. The lines extending from the central box represent the minimum and the maximum of the data.*

Please do not adjust margins



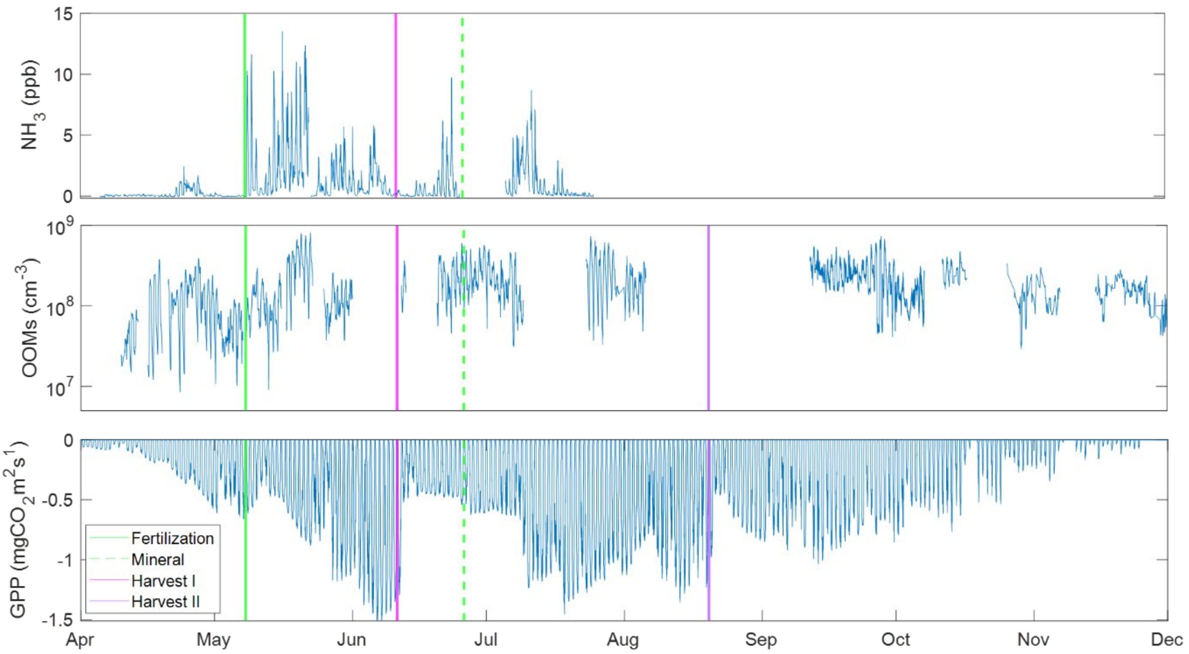


*Figure S 9 Agricultural land management during the year 2019 and its effect on ammonia ($NH_3$) – $NH_3$ was below detection limit after august, on total oxygenated organic molecules concentration (OOMs), and on gross primary production (GPP).*



Please do not adjust margins

Please do not adjust margins



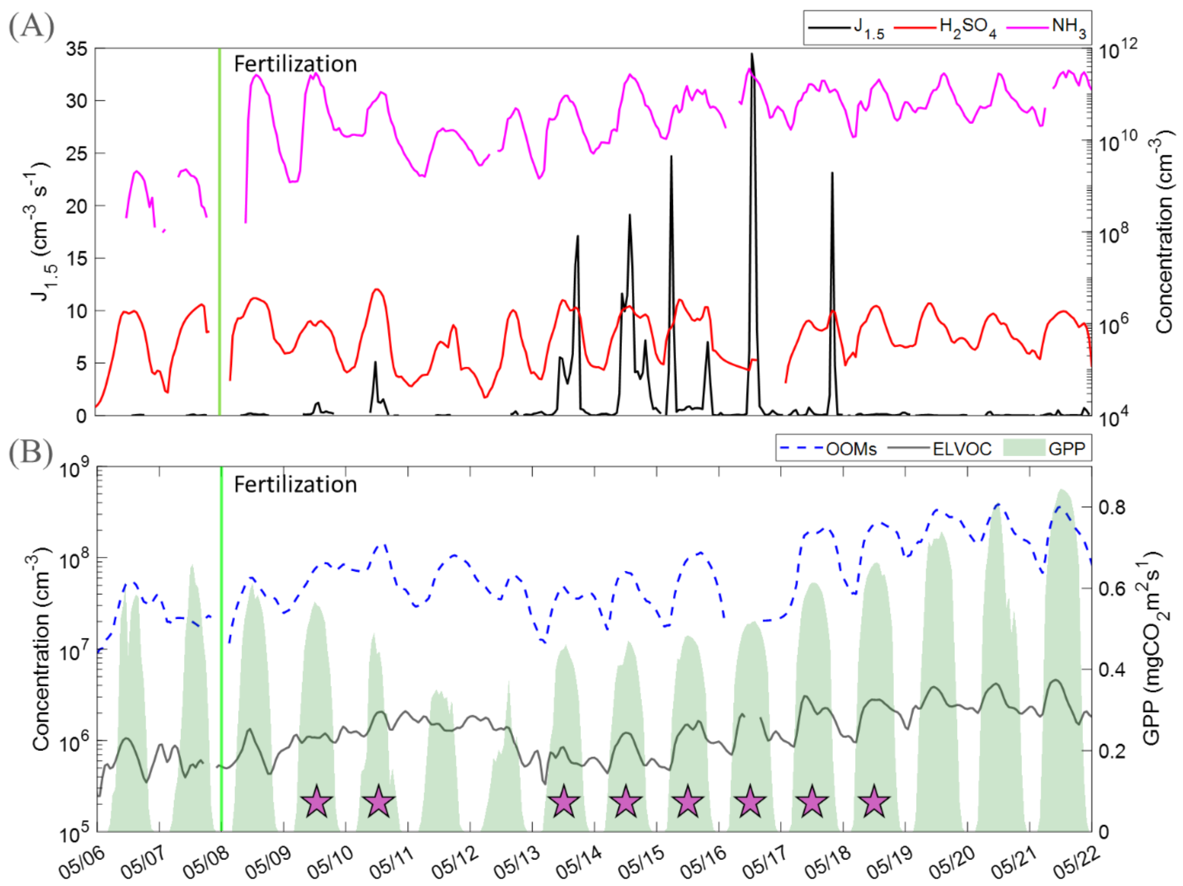


*Figure S 10 Excerpt of particle formation mechanism in Qvidja. (A) Time series of particle formation rates ($J_{1.5}$), and concentrations of sulfuric acid ($H_2SO_4$) and ammonia ($NH_3$). (B) Time series of gross primary production (-GPP) and concentration of oxygenated organic molecules (OOMs) and extremely low volatility organic compounds (ELVOC) during May 2019 in Qvidja. Clustering events are marked with a star. The fertilization day is marked with a green vertical line.*



This journal is © The Royal Society of Chemistry 20xx

Please do not adjust margins

Please do not adjust margins



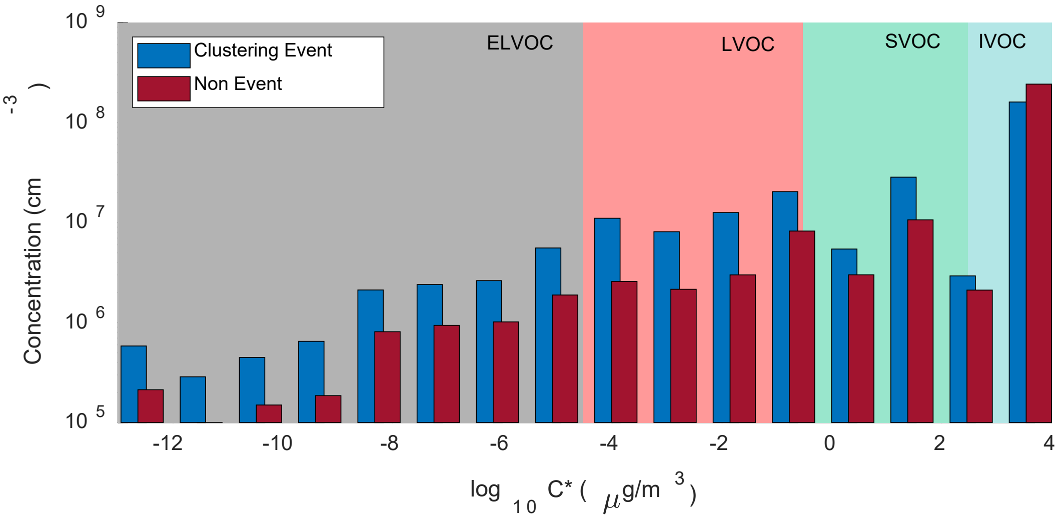


*Figure S 11 Volatility distribution bins of molecules measured on a local clustering event day on May 15, 2019 and on a non-event day May 6, 2019. See also the mass defect plots in Fig. 6. ELVOC, extremely low-volatility organic compound; IVOC, intermediate-volatility organic compound; LVOC, low-volatility organic compound; SVOC, semi-volatile organic compound.*



Please do not adjust margins
